# Layered Perovskites BaLn_n_In_n_O_3n+1_ (*n* = 1, 2) for Electrochemical Applications: A Mini Review

**DOI:** 10.3390/membranes13010034

**Published:** 2022-12-28

**Authors:** Nataliia Tarasova

**Affiliations:** 1 The Institute of High Temperature Electrochemistry of the Ural Branch of the Russian Academy of Sciences, 620066 Yekaterinburg, Russia; natalia.tarasova@urfu.ru; 2 Institute of Hydrogen Energy, Ural Federal University, 620075 Yekaterinburg, Russia

**Keywords:** Ruddlesden–Popper structure, oxygen-ion conductivity, proton conductivity, proton-conducting solid oxide fuel cells

## Abstract

Modern humanity is facing many challenges, such as declining reserves of fossil energy resources and their increasing prices, climate change and an increase in the number of respiratory diseases including COVID-19. This causes an urgent need to create advanced energy materials and technologies to support the sustainable development of renewable energy systems including hydrogen energy. Layered perovskites have many attractions due to their physical and chemical properties. The structure of such compounds contains perovskite layers divided by layers with different frameworks, which provide their properties’ features. Proton-conduction layered perovskites open up a novel structural class of protonic conductors, potentially suitable for application in such hydrogen energy devices as protonic ceramic electrolysis cells and protonic ceramic fuel cells. In this mini review, the special features of proton transport in the novel class of proton conductors BaLn_n_In_n_O_3n+1_ (*n* = 1, 2) with a layered perovskite structure are observed and general regularities are discussed.

## 1. Introduction

Modern humanity is facing many challenges, such as declining reserves of fossil energy resources and their increasing prices [1,2,3,4], climate change [5,6,7,8] and an increase in the number of respiratory diseases including COVID-19 [9,10]. This causes an urgent need to create advanced energy materials and technologies for supporting the sustainable development of renewable energy systems [11,12,13,14,15]. Hydrogen energy, due to eco-friendliness and high efficiency, has a high priority among other renewable energy systems [16,17,18,19,20,21,22]. The joining of forces in the fields of inorganic materials science and engineering for energy production has allowed the creation of such devices for hydrogen production as protonic ceramic electrolysis cells and devices for using hydrogen as a fuel for protonic ceramic fuel cells [23,24,25,26,27,28,29,30,31,32,33,34]. Among other materials, such devices need proton-conducting materials with a high level of proton conductivity and high resistance to carbon dioxide and water vapors [35].

**Figure 1:**
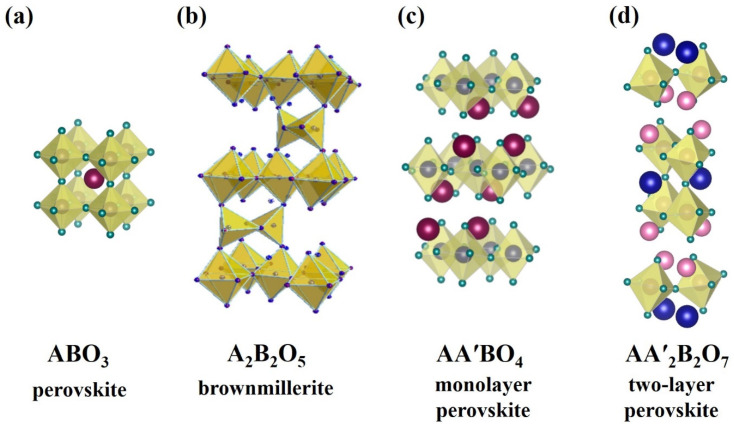
Crystal structures of perovskite (**a**), brownmillerite (**b**), monolayer (**c**) and two-layer (**d**) perovskites.

The classic materials investigated as protonic conductors for over forty years such as barium cerates and zirconates have a perovskite structure [36,37,38,39,40]. The realization of protonic conductivity in such types of materials is due to the dissociative intercalation of water molecules into a crystal lattice of complex oxides and subsequent proton transport. Consequently, an increase in proton concentration and mobility should lead to an increase in protonic conductivity in the general case:

(1) $$\sigma_{i}=z_{i}\cdot e\cdot c_{i}\cdot \mu_{i},\;$$

where *σ* is the electrical conductivity; *z* is the charge of current carriers; *c* and *μ* are their corresponding concentration and mobility. The possibility of water uptake for the complex oxides with the perovskite structure ABO_3_ (Figure 1a) is due to the creation of oxygen vacancies in the structure by the acceptor doping AB_1−*x*_M*_x_*O_3−δ_:

(2) $$V_{o}^{••}+H_{2}O+O_{o}^{\times }\Leftrightarrow 2{\left(\mathrm{OH}\right)}_{o}^{•},\;$$

where $V_{o}^{••}$ is the oxygen vacancy; $O_{o}^{\times }$ is the oxygen atom in the regular position; ${\left(\mathrm{OH}\right)}_{o}^{•}$ is the hydroxyl group in the oxygen sublattice. In this case, the oxygen vacancy concentration is strongly dependent on the acceptor dopant concentration $\left[V_{o}^{••}\right]=\text{½}\left[M_{B}^{\prime }\right]$ and it does not exceed 0.15–0.20 mol water per complex oxide formula unit.

**Figure 2:**
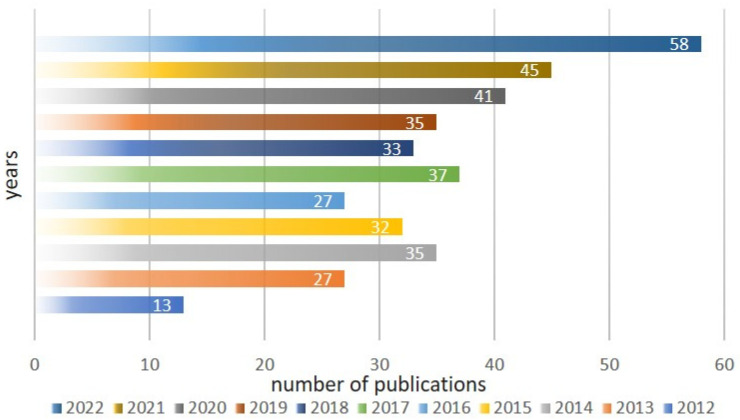
The progress of recent investigations into layered perovskites as prospective materials for solid oxide fuel cells.

The increase in the oxygen vacancies in the structure can be obtained not only by the modification of the matrix complex oxide structure but also by the principal change in the structural class of material. The acceptor-doped perovskites have an impure oxygen disorder, but the perovskite-related materials with their own structural oxygen disorders such as A_4_B_2_B′_2_O_11_ (for example Ba_4_Ca_2_Nb_2_O_11_ [41]) and A_2_B_2_O_5_ (Figure 1b) (for example Ba_2_In_2_O_5_ [42]) are known. The maximal water uptake is achieved for materials with the brownmillerite structure A_2_B_2_O_5_ ≡ ABO_2.5_ and it is 0.5 mol water per perovskite formula unit:

(3) $$V_{o}^{\times }+H_{2}O+2O_{o}^{\times }\Leftrightarrow 2{\left(\mathrm{OH}\right)}_{o}^{•}+O_{i}^{\prime \prime },\;$$

where $V_{o}^{\times }$ is the structural oxygen vacancy; $O_{o}^{\times }$ is the oxygen atom in the regular position; ${\left(\mathrm{OH}\right)}_{o}^{•}$ is the hydroxyl group in the oxygen sublattice; $O_{i}^{\prime \prime }$ is the oxygen atom in the interstitial position. Further, overcoming the limitation of the proton concentration in the complex oxide structure needs the next materials search among other perovskite-related structures. In the last decade, layered perovskites have been investigated as prospective materials for use in such electrochemical devices as solid oxide fuel cells (Figure 2), mostly however as electrode materials. In this paper, the details of proton transport in the novel class of proton conductors BaLn_n_In_n_O_3n+1_ (*n* = 1, 2) with a layered perovskite structure are observed and general regularities are discussed.

## 2. Structural Features of Layered Perovskite-Related Materials

In general, layered perovskites can be described as compounds containing perovskite layers divided by layers with different frameworks that provide their property features. Layered perovskites can be classified as Ruddlesden–Popper, Dion–Jacobson and Aurivillius structures. The last two are mostly represented by materials with photocatalytic [43,44,45,46,47,48,49,50,51,52,53], ferroelectric [54,55,56,57,58] and luminescent [59,60,61,62,63] properties. In this paper, we focused on the proton-conducing layered perovskites with a Ruddlesden-Popper structure, which represent a large novel class of proton-conducting materials. They can be described by the general formula A*_n_*_+1_B*_n_*O_3*n*+1_, in which rock salt layers AO and perovskite blocks (ABO_3_)*_n_* are contained. The alternation of rock salt layers and perovskite blocks was described for the first time by S.N. Ruddlesden and P. Popper with the examples of the Sr_2_TiO_4_ [64] and Sr_2_Ti_2_O_7_ [65] compounds about sixty-five years ago.

Some of crystal chemical criteria for the existence of layered Ruddlesden-Popper structures such as cation size ratio R¯ARB and bond iconicity I_AO_, where

(4) $$I_{\mathrm{AO}}=\frac{i_{\mathrm{AO}}}{i_{\mathrm{AO}}+i_{\mathrm{BO}}}$$

are highlighted [66,67,68]. The monolayer (*n* = 1) AA′BO_4_ compositions should have R¯ARB in the range 1.473–2.78, and I_AO_ between 0.523 and 0.628. The two-layer (*n* = 2) AA′_2_B_2_O_7_ compositions are characterized by a cation size ratio R¯ARB 1.606–2.262 and bond iconicity I_AO_ 0.519–0.624. In addition, the compositions must have tetragonal symmetry *I*_4_/*mmm* when the cation size ratio is higher than 1.75 and 1.87 for monolayer and two-layer structures, correspondingly.

Many monolayer AA′BO_4_ (Figure 1c) compositions including BaLaInO_4_ have the coordination formula $A^{\mathrm{IX}}A^{\prime \mathrm{IX}}B^{\mathrm{VI}}O_{4}^{\mathrm{VI}}$ [69]. However, the distortion of the crystal lattice leads to the possibility of other metal coordination numbers. For example, the BaNdInO_4_ composition is characterized by monoclinic symmetry and it has an $A^{\mathrm{XI}}A^{\prime \mathrm{VII}}B^{\mathrm{VI}}O_{4}^{\mathrm{VI}}$ coordination formula [70]. The two-layer perovskites (Figure 1d) have an $A^{\mathrm{XII}}A_{2}^{\prime \mathrm{IX}}B_{2}^{\mathrm{VI}}O_{7}^{\mathrm{VI}}$ coordination formula, and perovskite blocks consist of titled [BO_6_] octahedra [68]. This structure is characterized by the ordered arrangement of A- and A′-cations, which can be represented as [AO_12_] and [A′O_9_]. In the other words, the A-cations are located between [BO_6_] octahedra in the perovskite blocks, and the A′-cations are located in the rock salt layers. The ionic radius of the A′-cations is less than the A-cations, i.e., those kinds of atoms enter the inter-block space, whose ionic radius is lower. The decrease in the ionic radius of the rare-earth metal Ln for the layered perovskites $A^{\mathrm{II}}{\mathrm{Ln}}_{n}B_{n}^{\mathrm{III}}O_{3n+1}$ leads to the decrease in the space between perovskite blocks, i.e., to the decrease in the Ln–O2 bond and to the increase in [LnO_9_] polyhedra deformation. This is the reason for the increase in the destabilization of the layered perovskite structure and its approaching a classical perovskite structure.

**Figure 3:**
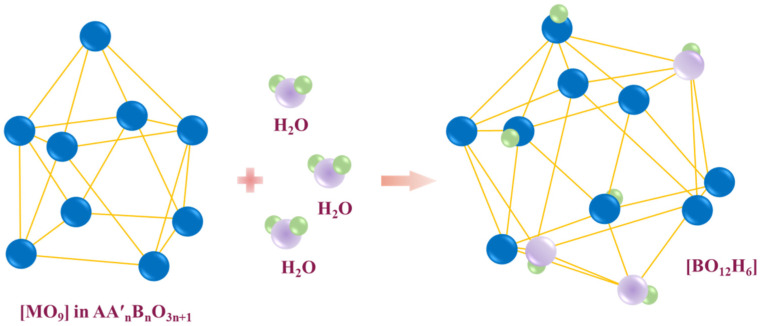
The schematic representation of the transformation of polyhedra [MO_9_] to [MO_12_] upon hydration.

The presence in the structure of layered perovskites the atoms of which can increase their coordination numbers ($A^{\mathrm{IX}}\to A^{\mathrm{XII}}$, for example) leads to the possibility of water uptake without the existence of oxygen vacancies (Figure 3). This process can be described as:

(5) $$H_{2}O+O_{O}^{x}\Leftrightarrow {(\mathrm{OH})}_{O}^{•}+{\left(\mathrm{OH}\;\right)}_{i}^{\prime },\;$$

where ${(\mathrm{OH})}_{O}^{•}$ is the hydroxyl group in the regular oxygen position; ${\left(\mathrm{OH}\;\right)}_{i}^{\prime }$ is the hydroxyl group located in the salt rock space. In the other words, the incorporated hydroxyl groups are placed into the salt block space. Consequently, the size of the salt block should play a significant role in the hydration properties of layered perovskites.

## 3. Oxygen-Ionic Transport in Layered Perovskite-Related Materials

The oxygen-ionic transport through the crystal lattice of layered perovskites can be described as the migration of oxygen point defects existing in the crystal due to anion Frenkel disorder:

(6) $$O_{o}^{\times }\;\;\Leftrightarrow V_{o}^{••}+O_{i}^{\prime \prime },\;$$

where $O_{o}^{\times }$ is the oxygen atom in the regular position; $V_{o}^{••}$ is the oxygen vacancy; $O_{i}^{\prime \prime }$ is the oxygen atom in the interstitial position. Doping (acceptor and donor) leads to changes in the oxygen stoichiometry, and hypostoichiometry and hyperstoichiometry are the result. Consequently, three possible oxygen migration mechanisms: (i) the direct interstitial mechanism, (ii) the interstitialcy mechanism and (iii) the vacancy mechanism can be considered [71]. The oxygen migration in monolayer perovskites where all elements have stable oxidation states is described by Yang et al. [72] and by Fujii et al. [73] for acceptor-doped compositions based on BaNdInO_4_. Both are proposed 2D oxygen ion diffusion mechanisms within rock salt layers. The mechanism of oxygen-ionic transport for the two-layer perovskite BaLn_2_In_2_O_7_ approved by calculation methods such as molecular dynamic simulation is not provided yet. However, we can suppose that ionic conductivity values must depend on the crystal structure features of layered compositions. The ratios of salt layers and perovskite blocks are 1:1 and 1:2 for monolayer and two-layer perovskites, correspondingly. Consequently, the ionic conductivity for two-layer perovskites must be lower in comparison with the conductivity for monolayer perovskites in the case of the realization of the 2D oxygen ion diffusion mechanisms for both. Oppositely, conductivity for the BaLn_2_In_2_O_7_ composition will be higher than for BaLnInO_4_ if the 3D transport mechanism is realized for the first.

**Figure 4:**
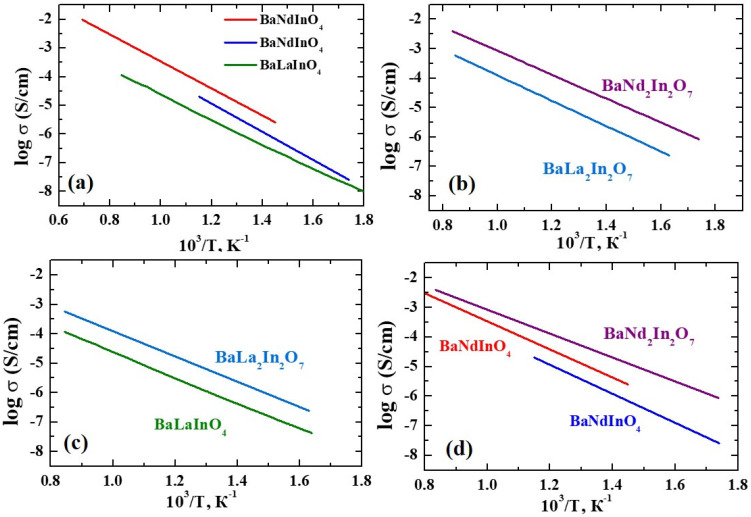
The temperature dependencies of electrical conductivity values obtained under dry air for monolayer BaLaInO_4_ [74], BaNdInO_4_ [70,71] compositions (**a**), two-layer BaLa_2_In_2_O_7_ [74] and BaNd_2_In_2_O_7_ [75] compositions (**b**), La-containing BaLaInO_4_ [74] and BaLa_2_In_2_O_7_ [74] compositions (**c**), Nd-containing BaNdInO_4_ [70,71] and BaNd_2_In_2_O_7_ [75] compositions (**d**).

Figure 4 represents the temperature dependencies of the electrical conductivity values obtained under dry air for the monolayers BaLaInO_4_ [74] and BaNdInO_4_ [70,71] and the two-layer BaLa_2_In_2_O_7_ [74] and BaNd_2_In_2_O_7_ [75] compositions. All of these samples were obtained by the solid-state method. The qualitative and quantitative compositions of the samples were confirmed by various methods such as XRD, SEM, EDS, EDX, etc. As can be seen, conductivity increases in the rows BaLaInO_4_-BaNdInO_4_ and BaLa_2_In_2_O_7_-BaNd_2_In_2_O_7_; i.e., Nd-containing compositions are more conductive than La-containing ones despite the number of *n* in the layered BaLn_n_In_n_O_3n+1_ structure. At the same time, two-layer compositions are more conductive than monolayer compounds. That is, the assumption about the 3D oxygen ion diffusion mechanism for two-layer perovskites BaLn_2_In_2_O_7_ is confirmed. 

The acceptor doping of the layered perovskites BaLn_n_In_n_O_3n+1_ can be described as:

(7) $$2\mathrm{AO}\overset{{\mathrm{Ln}}_{2}O_{3}}{\to }2A_{\mathrm{Ln}}^{\prime }+2O_{o}^{\times }+V_{o}^{••},\;$$

where $A_{\mathrm{Ln}}^{\prime }$ is the acceptor dopant in the sublattice of the rare-earth metal Ln; $O_{o}^{\times }$ is the oxygen atom in the regular position; $V_{o}^{••}$ is the oxygen vacancy. The appearance of oxygen vacancies in the crystal lattice of layered perovskites leads to the increase in conductivity values for both the monolayer BaLnInO_4_ and two-layer BaLa_2_In_2_O_7_ compositions. However, some features can be highlighted. Firstly, the increase in the conductivity values for the monolayer BaLaInO_4_ [76,77,78,79,80,81,82,83] and BaNdInO_4_ [70,72,73,84,85,86] compositions is more significant than for the two-layer BaLa_2_In_2_O_7_ [74,87,88,89,90] and BaNd_2_In_2_O_7_ [75,91] compositions (~1.5 vs. ~1. order of magnitude). Secondly, both the monolayer BaLnInO_4_ and two-layer BaLn_2_In_2_O_7_ compositions have different conductivity values with the same acceptor dopant concentration. Thirdly, the conductivity changes non-linearly with the increase in oxygen vacancy concentration. 

The donor doping of the layered perovskite BaLn_n_In_n_O_3n+1_ can be described as:

(8) $$2{\mathrm{MO}}_{2}\overset{{\mathrm{In}}_{2}O_{3}}{\to }2M_{\mathrm{In}}^{•}+3O_{o}^{\times }+O_{i}^{\prime \prime },\;$$

where $M_{\mathrm{In}}^{•}$ is the donor dopant in the indium sublattice; $O_{i}^{\prime \prime }$ is the oxygen atom in the interstitial position. As opposed to acceptor doping, donor doping can be realized only for the monolayer BaLnInO_4_ composition. The only mention of donor-doped two-layered composition was for BaLa_2_In_0.9_Ti_0.1_O_7.05_; however, the conductivity values for it were lower than for the undoped BaLa_2_In_2_O_7_ composition [78]. Consequently, it can be summarized that the crystal lattice of the two-layer BaLn_2_In_2_O_7_ perovskites is less tolerant to the presence of “additional” interstitial oxygen in comparison with the monolayer BaLnInO_4_ perovskites. The possibility of isovalent doping was proved for the monolayer and two-layer La-containing composition. The compositions with the doping of the La-sublattice BaLa_0.9_Gd_0.1_InO_4_ [81], BaLa_0.9_Nd_0.1_InO_4_ [82], BaLa_2–*x*_Gd*_x_*In_2_O_7_ 0 ≤ *x* ≤ 0.15 [90] and In-sublattice BaLaIn_0.9_Sc_0.1_O_4_ [79], BaLaIn_1−*x*_Y*_x_*O_4_ (0 ≤ *x* ≤ 0.5) [80] were obtained and investigated.

**Figure 5:**
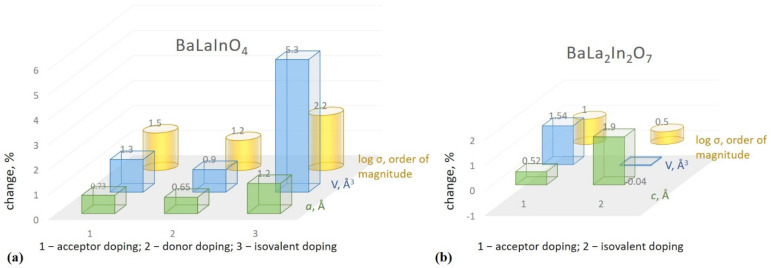
The changes in the lattice parameters, unit cell volumes and oxygen-ionic conductivity values for doped compositions based on monolayer BaLaInO_4_ (**a**) and two-layer BaLa_2_In_2_O_7_ perovskites (**b**).

The correlation between changes in the structural characteristics (lattice parameters, unit cell volumes) and conductivity values for doped compositions based on BaLaInO_4_ and BaLa_2_In_2_O_7_ is presented in Figure 5. The increase in the unit cell volume for monolayer compositions is due to the increase in the *a* lattice parameter, which indicates the increase in the distance between perovskite blocks. In general, this increase correlates well with the increase in electrical conductivity values upon doping. It allows us to say that the geometric factor has a significant effect on the change in electrical conductivity. This is also a key factor in understanding why related compositions have different conductivity at the same concentration of oxygen vacancies. It should be noted that the reasons for the increase in lattice parameters are different for each type of doping. The introduction of acceptor dopants with bigger ionic radii (Sr/Ba → La), the appearance of “additional” interstitial oxygen during donor doping and the appearance of the additional repulsion effects of the different nature ions in one sublattice during isovalent doping can be named as the more significant reasons for the increase in the *a* lattice parameter. 

For two-layer perovskites based on BaLa_2_In_2_O_7_, the clearer correlation is between the *c* lattice parameter, which indicates the increase in the distance between perovskite blocks for this type of crystal structure, and conductivity values. As it was for monolayer compositions, the increase in the distance between perovskite blocks leads to the increase in the conductivity values. However, this increase is smaller, which can be explained by different conduction mechanisms for each type of layered structure.

Despite the increase in distance between perovskite blocks during heterovalent (donor and acceptor) doping, conductivity values increase at the “small” dopant concentrations (i.e., at “small” oxygen point defect ($V_{o}^{••}$ or $O_{i}^{\prime \prime }$) concentrations) and decrease at the “big” concentrations. The most probable reason is the formation of clusters with lower mobility and a decrease in the concentration of mobile oxygen defects. The defect association can describe the acceptor type of doping as:

(9) $$M_{\mathrm{Ln}}^{\prime }+V_{o}^{••}\to {\left(M_{\mathrm{Ln}}^{\prime }\cdot V_{o}^{••}\right)}^{•}$$

or

(10) $$M_{\mathrm{Ln}}^{\prime }+{\left(M_{\mathrm{Ln}}^{\prime }\cdot V_{o}^{••}\right)}^{•}\to {\left(2M_{\mathrm{Ln}}^{\prime }\cdot V_{o}^{••}\right)}^{\times },\;$$

and for the donor type of doping as

(11) $$M_{\mathrm{In}}^{•}+O_{i}^{\prime \prime }\to {\left(M_{\mathrm{In}}^{•}\cdot O_{i}^{\prime \prime }\right)}^{\prime }$$

or

(12) $$M_{\mathrm{In}}^{•}+{\left(M_{\mathrm{In}}^{•}\cdot O_{i}^{\prime \prime }\right)}^{\prime }\to {\left(2M_{\mathrm{In}}^{•}\cdot O_{i}^{\prime \prime }\right)}^{\times }$$

It is clear that both factors, the increase in the lattice parameters (geometric factor) and the increase in the oxygen defects concentration (concentration factor), affect the electrical conductivity value simultaneously. Consequently, the exclusion of one of these factors can help determine the most significant cause of changes in the electrical conductivity values. Both heterovalent doping and isovalent doping lead to an increase in the lattice parameters, but the oxygen concentration does not change.

**Figure 6:**
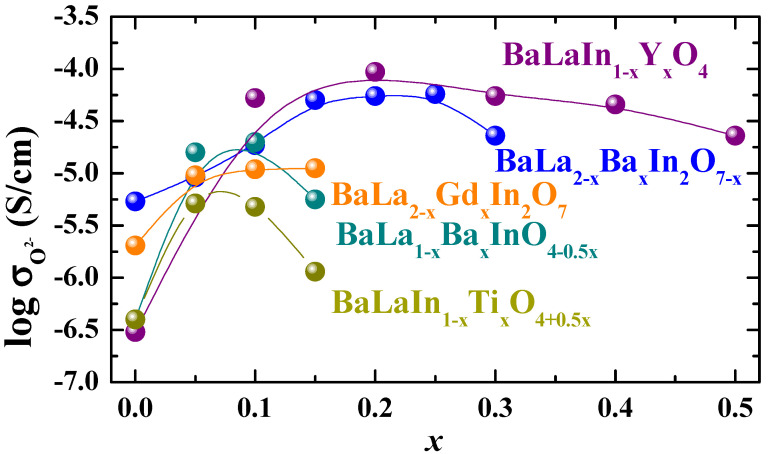
The concentration dependencies of oxygen-ionic conductivities for heterovalent-doped BaLa_1–*x*_Ba*_x_*InO_4–0.5*x*_ [76], BaLaIn_1–*x*_Ti*_x_*O_4+0.5*x*_ [78], BaLa_2–*x*_Ba*_x_*In_2_O_7–*x*_ [89] and isovalent-doped BaLaIn_1−*x*_Y*_x_*O_4_ [80], BaLa_2–*x*_Gd*_x_*In_2_O_7_ [91] compositions at 500 °C.

Figure 6 represents the concentration dependencies of the oxygen-ionic conductivities for heterovalent-doped BaLa_1–*x*_Ba*_x_*InO_4–0.5*x*_ [76], BaLaIn_1–*x*_Ti*_x_*O_4+0.5*x*_ [78], BaLa_2–*x*_Ba*_x_*In_2_O_7–*x*_ [89] and isovalent-doped BaLaIn_1−*x*_Y*_x_*O_4_ [80], BaLa_2–*x*_Gd*_x_*In_2_O_7_ [91] compositions. As can be seen, isovalent doping allows the obtaining of more conductive compositions at the same dopant concentration for monolayer perovskites based on BaLaBaInO_4_. Conversely, acceptor doping is a more suitable way to increase the conductivity values of two-layered perovskites compared with isovalent doping. The crystal lattice of monolayer BaLnInO_4_ perovskites is more flexible than two-layer BaLa_2_In_2_O_7_ perovskites, which allows for a more significant increase in the distance between perovskite blocks and in the conductivity values. However, the decrease in the conductivity values at a “big” acceptor dopant concentration is more valuable for monolayer BaLa_1–*x*_Ba*_x_*InO_4–0.5*x*_ perovskites compared with two-layer BaLa_2–*x*_Ba*_x_*In_2_O_7–*x*_. We can suppose that the reason for this difference is the change in the ratio of perovskite blocks and salt layers and in the mechanism of oxygen transport. 

## 4. Proton Transport in Layered Perovskite-Related Materials

**Figure 7:**
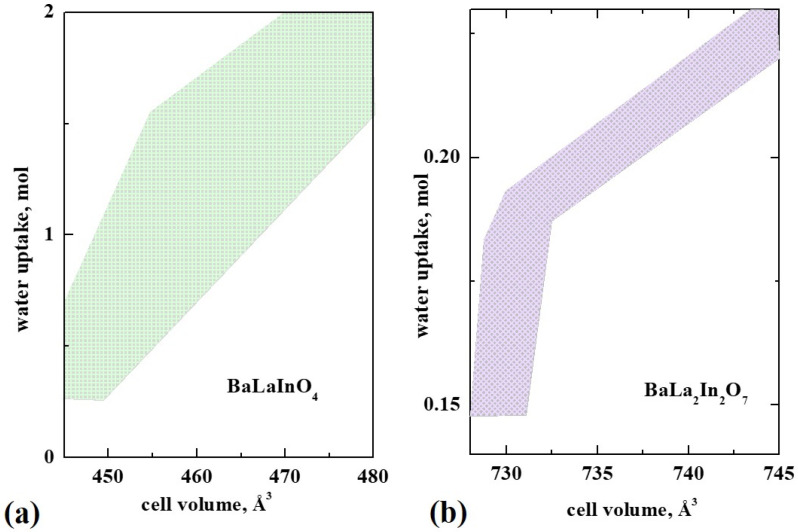
The dependencies of water uptake for doped compositions based on BaLaInO_4_ monolayer (**a**) and two-layer BaLa_2_In_2_O_7_ (**b**) perovskites.

Protonic conductivity directly depends on the proton concentration in the structure (Equation (1)), and the possibility of water uptake for layered perovskites is very important. The possibility for water intercalation for undoped and doped layered BaLaInO_4_ perovskites was proved, and the absence of correlation between oxygen vacancy concentration and water uptake was shown [77]. Figure 7a represents the dependency of water uptake vs. unit cell volume for monolayer perovskites. As we can see, the water uptake for doped perovskites based on BaLaInO_4_ is much higher than for classical ABO_3_ perovskites (up to ~2 mol water per complex oxide formula unit). In addition, the water uptake for isovalent-doped compositions is higher in comparison with isovalent-doped compositions. The possible reason is the absence of “additional” oxygen point defects ($V_{o}^{••}$ or $O_{i}^{\prime \prime }$) in the salt layer space, which can make the dissociative intercalation of water molecules into the crystal lattice difficult.

The dependency of water uptake vs. unit cell volume for two-layer perovskites is presented in the Figure 7b. Contrary to monolayer perovskites, the changes in the unit cell volume do not significantly affect the amount of water uptake. It slightly increases with the increase in oxygen concentration, but it does not reach the maximum possible values equal to the concentration of vacancies. In general, the water uptake for all undoped and doped two-layer perovskites based on BaLn_2_In_2_O_7_ is about 0.10–0.22 mol per complex oxide formula unit [87], which is comparable with the values for acceptor-doped classic perovskites.

**Figure 8:**
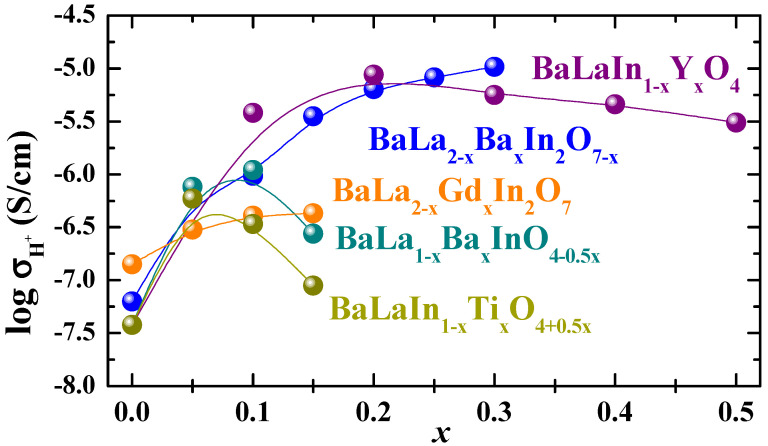
The concentration dependencies of protonic conductivities for heterovalent-doped BaLa_1–*x*_Ba*_x_*InO_4–0.5*x*_ [76], BaLaIn_1–*x*_Ti*_x_*O_4+0.5*x*_ [78], BaLa_2–*x*_Ba*_x_*In_2_O_7–*x*_ [86] and isovalent-doped BaLaIn_1−*x*_Y*_x_*O_4_ [80], BaLa_2–*x*_Gd*_x_*In_2_O_7_ [90] compositions at 300 °C.

The concentration dependencies of the proton conductivity values for monolayer and two-layer compositions are presented in Figure 8. As can be seen, the general regularities of protonic conductivity are similar to the regularities of oxygen-ionic conductivity (Figure 6). The decrease in the proton conductivity at a “big” dopant concentration is observed for heterovalent-doped monolayer perovskites based on BaLaInO_4_. The cluster formation can be named as the reason for this decrease and it can be expressed by the following equations:

(13) $$M_{\mathrm{Ln}}^{\prime }+{(\mathrm{OH})}_{o}^{•}\to {\left(M_{\mathrm{Ln}}^{\prime }\cdot {(\mathrm{OH})}_{o}^{•}\right)}^{\times }$$

(14) $$M_{\mathrm{In}}^{•}+{(\mathrm{OH})}_{i}^{\prime }\to {\left(M_{\mathrm{In}}^{•}\cdot {(\mathrm{OH})}_{i}^{\prime }\right)}^{\times }$$

The greatest increase in conductivity is achieved by isovalent doping for monolayer compositions and by acceptor doping for two-layer compositions. The comparison of the protonic conductivity values of layered perovskites BaLn_n_In_n_O_3n+1_ (*n* = 1, 2) is presented in Table 1. The conductivity values for most conductive monolayer BaLaIn_1−*x*_Y*_x_*O_4_ and two-layer BaLa_2–*x*_Ba*_x_*In_2_O_7–*x*_ compositions are comparable. This allows us to say that a significant decrease (about one order of magnitude) in the proton concentration for two-layer perovskites compared to monolayer perovskites is compensated for by the increase in their mobility. Obviously, this increase is due to the increase in oxygen-ion mobility since the transfer of protons is carried out by moving along oxygen ions. We can suggest that the inclusion of perovskite blocks in the crystal lattice (two-layer perovskites) and the creation of oxygen vacancies into them (acceptor doping) change the transport mechanism from 2D to 3D and significantly increase the ionic conductivity values. In general, an increase in the number of perovskite stacks in the blocks of the layered structure leads to an increase in the ionic conductivity values of the layered perovskites BaLn_n_In_n_O_3n+1_ (*n* = 1, 2).

One of the next steps for using layered perovskites for electrochemical applications is the transition from material creation to manufacturing technology. In this regard, the problem of comparability between materials of different device components such as electrode and electrolyte materials becomes very important. We believe that the investigation of stacks where both electrode and electrolyte materials have a layered structure will happen in the near future.

## 5. Conclusions

In this paper, the specifics of proton transport in the novel class of proton conductors BaLn_n_In_n_O_3n+1_ (*n* = 1, 2) with a layered perovskite structure are observed and general regularities are discussed. The conductivity values are strongly dependent on the nature of the rare-earth metal Ln and of a number of perovskite *n* stacks in the structure. The Nd-containing compositions are more conductive than the La-containing ones at the same *n*. When the nature of the rare-earth metal does not change, two-layer perovskites have higher conductivity values in comparison with monolayer compounds. The crystal lattice of monolayer perovskites is more “flexible” and allows the performance of various types of doping such as acceptor, donor and isovalent doping, while donor doping is not suitable for the two-layer perovskite structure. The water uptake for doped monolayer perovskites is much higher than for classical ABO_3_ perovskites (up to ~2 mol water per complex oxide formula unit) and it is comparable (~0.2 mol water) for two-layer perovskites. The greatest increase in conductivity is achieved by isovalent doping for monolayer compositions and by acceptor doping for two-layer compositions. At the same time, the conductivity values for most conductive monolayer BaLaIn_1−x_Y_x_O_4_ and two-layer BaLa_2–*x*_Ba*_x_*In_2_O_7–*x*_ compositions are comparable.

We can conclude that the increase in the number of perovskite stacks in the blocks of the layered structure approaches the properties of the layered perovskite closer to classical perovskite. The ionic conductivity values of layered perovskites BaLn_n_In_n_O_3n+1_ (*n* = 1, 2) increase with increasing *n*, but the water uptake decreases. At the same time, layered perovskites are a prospective class due to their structural “flexibility”, ability to vary the concentration of protons and conductivity over a wide range (up to 2 orders of magnitude) during doping. The optimal combination of cations and doping method will allow the obtaining of novel high-conductive materials suitable for application as protonic electrolytes in different electrochemical devices, and the next materials search will be very relevant.

## Figures and Tables

**Table 1:** The comparison of the protonic conductivity values of layered perovskites BaLn_n_In_n_O_3n+1_ (*n* = 1, 2).

Composition	Values of Protonic Conductivity at 450 °C, S/cm	Ref.
BaLaInO_4_	4 × 10^−7^	[83]
Ba_1.1_La_0.9_InO_3.95_	0.8 × 10^−5^	[83]
BaLa_0.9_Sr_0.1_InO_3.95_	1.4 × 10^−5^	[83]
BaLa_0.9_Ca_0.1_InO_3.95_	1.9 × 10^−5^	[83]
BaLaIn_0.9_Ti_0.1_O_4.05_	0.2 × 10^−5^	[78]
BaLaIn_0.9_Zr_0.1_O_4.05_	0.2 × 10^−5^	[78]
BaLa_0.9_Nd_0.1_InO_4_	1.1 × 10^−5^	[82]
BaLa_0.9_Gd_0.1_InO_4_	1.0 × 10^−5^	[81]
BaLaIn_0.1_Y_0.9_O_4_	3.3 × 10^−5^	[80]
BaLaIn_0.1_Sc_0.9_O_4_	1.2 × 10^−5^	[79]
BaLa_2_In_2_O_7_	1.7 × 10^−6^	[75]
BaLa_0.9_Ca_0.1_InO_3.95_	2.3 × 10^−6^	[74]
Ba_1.05_La_1.95_In_2_O_6.975_	5.4 × 10^−6^	[87]
Ba_1.1_La_1.9_In_2_O_6.95_	10 × 10^−6^	[87]
Ba_1.2_La_1.8_In_2_O_6.9_	63 × 10^−6^	[87]
Ba_1.25_La_1.75_In_2_O_6.875_	64 × 10^−6^	[87]
BaLa_1.9_Sr_0.1_In_2_O_6.95_	9.4 × 10^−6^	[87]
BaLa_1.85_Sr_0.15_In_2_O_6.925_	14 × 10^−6^	[87]
BaLa_1.8_Sr_0.2_In_2_O_6.9_	17 × 10^−6^	[87]
BaLa_1.95_Gd_0.05_In_2_O_7_	1.8 × 10^−6^	[90]
BaLa_1.9_Gd_0.1_In_2_O_7_	2.3 × 10^−6^	[90]
BaLa_1.85_Gd_0.15_In_2_O_7_	2.7 × 10^−6^	[90]

## Data Availability

Not applicable.
